# The potential of remote sensing for improved infectious disease ecology research and practice

**DOI:** 10.1098/rspb.2024.1712

**Published:** 2024-12-18

**Authors:** Claire S. Teitelbaum, António Ferraz, Susan E. W. De La Cruz, Morgan E. Gilmour, Ian G. Brosnan

**Affiliations:** ^1^ NASA Ames Research Center, Moffett Field, CA, USA; ^2^ Bay Area Environmental Research Institute, Moffett Field, CA, USA; ^3^ U.S. Geological Survey, Western Ecological Research Center, San Francisco Bay Estuary Field Station, Moffett Field, CA, USA; ^4^ NASA Jet Propulsion Laboratory, Pasadena, CA, USA

**Keywords:** infectious disease ecology, pathogens, remote sensing, soil moisture, surface water, vegetation

## Abstract

Outbreaks of COVID-19 in humans, Dutch elm disease in forests, and highly pathogenic avian influenza in wild birds and poultry highlight the disruptive impacts of infectious diseases on public health, ecosystems and economies. Infectious disease dynamics often depend on environmental conditions that drive occurrence, transmission and outbreaks. Remote sensing can contribute to infectious disease research and management by providing standardized environmental data across broad spatial and temporal extents, often at no cost to the user. Here, we (i) conduct a review of primary literature to quantify current uses of remote sensing in disease ecology; and (ii) synthesize qualitative information to identify opportunities for further integration of remote sensing into disease ecology. We identify that modern advances in airborne remote sensing are enabling early detection of forest pathogens and that satellite data are most commonly used to study geographically widespread human diseases. Opportunities remain for increased use of data products that characterize vegetation, surface water and soil; provide data at high spatio-temporal and spectral resolutions; and quantify uncertainty in measurements. Additionally, combining remote sensing with animal telemetry can support decision-making for disease management by providing insights into wildlife disease dynamics. Integrating these opportunities will advance both research and management of infectious diseases.

## 1. Introduction

Understanding the ecology of infectious diseases is critical for forecasting and managing disease impacts on public health, wildlife management, sustainability and agriculture [1–3] and for ecology and biodiversity science more broadly. Not only can diseases regulate population sizes of their hosts, which scale up to affect ecosystem processes, but pathogens and parasites (hereafter ‘pathogens’) also form a significant portion of global biomass [4]. The dynamics of most infectious diseases rely in part on environmental conditions. Pathogens that persist in the environment can be sensitive to temperature, humidity and pH. Pathogen transmission also depends on host distributions and behaviour, which in turn are determined by habitat and climatic conditions. Infectious disease ecology research therefore requires both a detailed understanding of the mechanisms driving disease dynamics and a broad-scale understanding of pathogen–environment relationships at global and decadal scales.

Satellite and airborne remote sensing, which involves Earth observations without interacting with objects, are essential tools for measuring environmental conditions at spatiotemporal scales relevant to infectious disease ecology research and management. Remotely sensed data can provide repeated standardized measurements across extensive spatial and temporal extents; are automated (i.e. require only limited field effort after calibration and ground-truthing are complete); and can measure remote areas of the globe (e.g. open ocean, tropical forests). Remote sensing can also provide retrospective data on conditions preceding a disease outbreak [5]. Ever-advancing technologies for remote sensing are providing higher spatial and temporal resolution data on a growing number of biotic and abiotic variables. These advances might be particularly important in regions where scientists work with relatively limited resources; freely available remote sensing data are associated with increased scientific output from middle- and low-income nations across multiple scientific fields [6].

As in other fields of applied ecology, remote sensing has contributed to important advances in disease ecology [7–9]. For example, early warning systems for cholera based on remotely sensed sea surface temperature and salinity allow public health entities to issue warnings before the first human case is detected [10]. However, opportunities remain for better integration of existing remote sensing data in disease research as well as the development of disease-relevant remote sensing products. Here, we conduct a quantitative review of primary literature to understand how remote sensing data have been used in infectious disease ecology, then use this information to identify existing and future opportunities for disease ecologists to use remote sensing, including new or planned sensors, underused existing data and methods or systems that are amenable to the use of remote sensing data.

## 2. Quantitative review

### (a) Methods

We searched Web of Science™ on 30 March 2023 with the following string:

(‘remote* sens*’ OR satellite OR imag* OR ‘laser scan*’ OR LIDAR OR ‘light detection and ranging’ OR thermal OR infrared OR multispectral OR hyperspectral OR RGB OR ‘red green blue’ OR raster* OR ‘digital elevation model*’ OR ‘digital terrain model*’ OR ‘digital surface model*’ OR ‘elevation model*’ OR ‘digital terrain’ OR DEM OR DTM OR DSM OR DEMs OR DTMs OR DSMs OR drone OR UAV OR ‘aerial vehicle’ OR ‘aerial system’) AND ecolog* AND (disease* OR infect* OR zoono* OR parasit* OR pathogen)

This search was designed to find ecological studies of disease that used remote sensing data from satellites and airborne platforms, including uncrewed aerial vehicles (UAVs), but excluding field imagery such as cameras installed at field sites. Web of Science™ covers both environmental science and public health [11]. We searched a single database because we aimed to identify general patterns and knowledge gaps, rather than to answer a specific question, for which use of a single database is appropriate [12,13].

The search returned 4022 potentially relevant articles. We further screened abstracts and text, considering articles relevant if they:

- used remotely sensed data, as defined above;
- studied an infectious disease; and
- presented primary results (i.e. no review articles or meta-analyses).

Studies of invasive species, plant herbivores, parasitoids and non-infectious diseases were excluded but studies of disease vectors or hosts without any disease or pathogen data were included if a focal disease was mentioned in the introduction section. Common reasons for exclusion were false hits from the terms ‘satellite’ (e.g. microsatellite loci) and ‘drone’ (e.g. honeybee studies), non-infectious diseases, and use of only *in situ* environmental data (e.g. weather stations, field surveys).

Next, we extracted data on hosts, pathogens, diseases, geographical locations, remote sensing data and methods from each article (table 1). For studies examining multiple hosts, diseases, and/or using multiple remote sensing products, we recorded all information meeting the criteria above. We extracted data from articles in English and French and used translation software (Google Translate™) to extract data from articles in other languages. A single reviewer screened abstracts and extracted data.

**Table 1.** Data extraction categories and variables recorded. (Each article usually produced multiple lines of data (e.g. multiple hosts, multiple remote sensing products).)

category	variables recorded
**host(s**)	— scientific name — common name — host role (host, accidental host, intermediate host, or vector) — host type (animal, plant, human; domestic, or wild)
**disease(s**)	— pathogen species (scientific name) — disease name — transmission mode(s) (e.g. environmental, vector, direct)
**location**	— continent, country and locality. Studies examining five or more of a category were marked as ‘global,’ ‘continental,’ or ‘multiple,’ respectively.
**remotely sensed data**	— sensor platform (satellite, airborne, or UAV) — sensor or dataset name (e.g. MODIS) — variable (e.g. land surface temperature) — spatial resolution — temporal resolution used (can differ from temporal resolution of product) — product source (source, other article, or this article).
**methods**	— disease data collection method (e.g. case reports, surveillance, field sampling) — disease data type (disease, pathogen, antibody, or none) — primary goals (e.g. mapping, explanatory, predictive, methods development) — use of species distribution modelling (yes or no)

### (b) Results and discussion

#### (i) Identified uses of remote sensing data

We identified 497 infectious disease ecology studies that used remote sensing data [14]. Uses of remote sensing data ranged from a single remotely sensed image used to identify study areas (e.g. household locations [15]) to developing new methods to map mosquito habitats from multi-sensor remote sensing data [16]. Most studies (64%) were classified as explanatory (i.e. seeking to understand correlates of disease occurrence) and/or mapping (i.e. seeking to determine spatial patterns in disease risk; 30%). Other common goals were methods development (e.g. statistical modelling methods; 16%), prediction (i.e. seeking to estimate disease occurrence outside the spatial or temporal range of data collection; 11%) and detection (i.e. using remote sensing to detect disease; 7%).

#### (ii) Use of remotely sensed variables across hosts and diseases

We classified remote sensing data by variable type (e.g. temperature, land cover, vegetation properties), source (i.e. satellite, airborne, UAV, or satellite + *in situ*) and horizontal spatial resolution (order of magnitude: <1 m, 1–10 m, 10–100 m, …). We classified diseases based on their transmission mode (vector, environmental, direct, airborne, complex and/or other) and their definitive host type(s) (human, livestock, domestic animal, wild animal, agricultural plant and/or wild plant).

**Figure 1.**
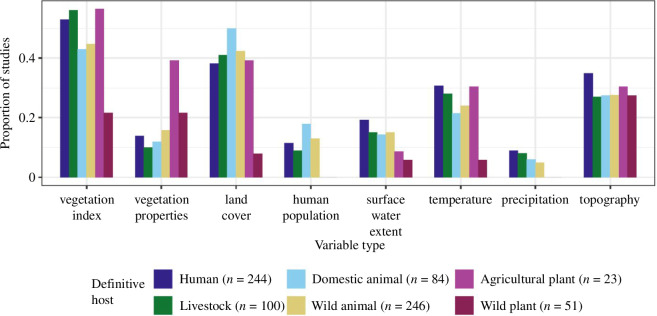
Use of remote sensing variables across diseases with different host types. The *y*-axis measures proportion of studies of diseases with each definitive host type that used at least one variable of the focal type (e.g. approx. 51% of human disease studies used at least one vegetation index). Note the absence of precipitation and human population data in studies of plant diseases and the over-representation of plant studies in the vegetation properties category. The eight most common variable types are shown and variables are grouped thematically. Numbers in the legend indicate the total number of studies with each definitive host type; studies are counted multiple times if they study a disease with multiple definitive host types (e.g. zoonoses).

The most commonly used remotely sensed variables were vegetation indices (e.g. normalized difference vegetation index; *n* = 239 studies), land cover (*n* = 180), topography (e.g. elevation, slope; *n* = 147), temperature (most often land surface temperature; *n* = 137), surface water extent (most often water body locations; *n* = 77) and vegetation properties (e.g. forest cover, canopy height; *n* = 76) (electronic supplementary material, figure S1A). Vegetation indices and topographical variables were commonly used across host types and transmission modes, but variables measuring vegetation properties were most common in studies of plant disease (figure 1). Conversely, precipitation and human population data were more commonly used in studies of animal diseases than for plant diseases (figure 1). These contrasting patterns probably indicate researchers’ assessment of epidemiological importance (e.g. human population density contributes directly to transmission of human diseases) as well as the availability of sufficiently detailed information. For example, many land cover data layers are resolved only to the level of ‘croplands’ or ‘forests’, in which case a study of a crop or forest disease might contain only a single land cover class.

Use of precipitation data (*n* = 35 studies) was more common in studies of vector-borne diseases than for other transmission modes, whereas surface water variables were most common in studies of pathogens with complex transmission modes (electronic supplementary material, figure S2). This pattern probably represents the importance of small ephemeral waterbodies as breeding habitat for malaria vectors, specifically *Anopheles* mosquitoes, whereas for diseases with complex transmission modes (e.g. dracunculiasis, schistosomiasis), human hosts are exposed via larger and/or permanent water bodies used for drinking, bathing and recreation [17,18]. These water bodies vary less in size and suitability in response to precipitation, making direct measurements of surface water more appropriate for permanent water bodies and, by extension, for diseases with complex transmission ([17,19]; but see [20] for similarities between mosquito and snail habitats).

#### (iii) Data sources and resolutions

Across host and disease types, most remote sensing data were derived from satellite sources; 86% of studies used at least one satellite-derived variable. The primary exception to this trend was for disease detection, which was primarily derived from airborne and UAV platforms. Since almost all disease detection focused on plant diseases (e.g. leaf colour changes), airborne- and UAV-sourced data were more common in the study of plant diseases than in diseases of animal hosts (electronic supplementary material, figure S3).

Most remote sensing products were at high to moderate spatial resolution (10–100 m and 100 m to 1 km), with resolutions as fine as 30 cm and as coarse as 275 km (electronic supplementary material, figure S1B). Airborne and UAV data were usually at finer spatial resolutions than satellite data, as were studies focusing on disease detection (which, accordingly, were more likely to use airborne and UAV data sources). By contrast, weather variables (i.e. temperature, precipitation) had the coarsest average spatial resolutions. These variables were often derived from products that used satellite data in combination with on-the-ground sensors (e.g. reanalysis products), indicating that these additional sensors were used to estimate variables that are not directly sensed by satellites. Data at scales finer than 1 m were dominated by plant studies, and no plant studies used variables with resolutions coarser than 1 km. Conversely, studies of wild animals rarely used variables with resolution finer than 10 m, possibly reflecting the relatively high mobility and relatively low population density of wild animals.

#### (iv) Study systems: diseases and hosts

**Figure 2.**
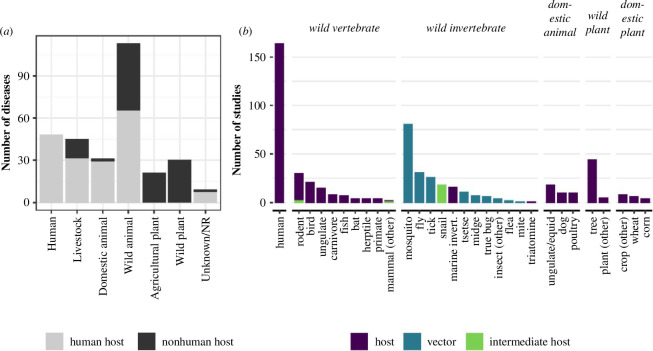
Study effort across host types. (*a*) Number of diseases with each definitive host type. Diseases with multiple definitive hosts can appear in multiple columns. Numbers differ from figure 1 because they count unique diseases, not studies. Some diseases of humans (grey) have humans as accidental hosts, not definitive hosts. Most diseases in all categories except plant diseases are zoonotic, i.e. infecting both wild animals and humans. (*b*) Study effort for individual host taxa. This metric considers the focal host(s) of a study rather than the definitive host of a disease. Studies are counted multiple times if they use data from multiple hosts.

Of the 497 studies identified in our review, the majority (*n* = 378, 76%) studied at least one disease relevant to human health. Most of these diseases were zoonotic, i.e. shared between humans and domestic or wild vertebrates (figure 2*a*). Malaria was the most studied disease (*n* = 67 studies), followed by leishmaniasis (*n* = 34), Dengue fever (*n* = 25), West Nile fever (*n* = 23), schistosomiasis (*n* = 22), hantavirus-caused diseases (*n* = 19) and avian influenza (*n* = 19; electronic supplementary material, figure S4). These diseases share several characteristics: they infect human hosts, have a non-human definitive host or are vector-borne, have high research effort and have relatively high incidence [21,22]. These patterns suggest that remote sensing might be especially useful for geographically widespread diseases with high incidence (although this is confounded by research effort, see below).

Neglected tropical diseases (NTDs), defined and enumerated by the World Health Organization as diseases that are ‘mainly prevalent among impoverished communities in tropical areas’ [23], comprised 26% of studies (*n* = 129), including some of the most-studied diseases (i.e. leishmaniasis, Dengue fever and schistosomiasis). The high prevalence of NTDs in the dataset could stem from a combination of their ecology and the strengths of remote sensing data. Dynamics of most NTDs are linked to the environment [23,24], and they often occur in rural areas that might be difficult to access, making remote sensing a potentially important tool for their study [25]. In addition, many NTDs have established prevention and control strategies, but resources for distributing these measures are limited [24], so mapping disease risk could be especially valuable for prioritizing interventions.

Among the studies that examined non-zoonotic and non-human diseases (*n* = 156, 25%), the most-studied diseases were pine wilt disease (*n* = 12 studies), sudden oak death (*n* = 11), white syndromes (a group of coral diseases, *n* = 8) and bluetongue (a disease of wild and domestic ruminants, *n* = 7). This relative dominance of research on plant diseases, sessile animals (corals) and livestock could be owing to the challenges inherent in sampling wildlife hosts to acquire disease data; compared to plants and livestock, wildlife are difficult to capture and infection prevalence is often low [26]. Accordingly, the most studied diseases of non-sessile wildlife were avian influenza (*n* = 19; sometimes zoonotic) and avian malaria (*n* = 5), both of which occur at relatively high prevalence and in multiple host species, making them easier to detect. However, study effort using remote sensing was still low in several important and relatively well-studied wildlife diseases that have close associations with environmental conditions, such as chytridiomycosis in amphibians (*n* = 3) and white-nose syndrome in bats (*n* = 1) [27,28].

Human diseases were the most numerous in our dataset, but they were also relatively well studied in the general ecological literature. When compared to the study effort for the ecology of each disease (gathered from an independent search of Web of Science™ that excluded remote sensing keywords), remote sensing studies made up 2.5–5% of studies of each of the six most-studied human diseases (electronic supplementary material, figure S5). By contrast, the diseases with the highest relative remote sensing study effort all had non-human hosts: sudden oak death, pine wilt disease and white syndromes (all > 7.5% remote sensing).

## 3. Contributions of remote sensing to disease ecology: current status and future opportunities

In this section, we summarize five common applications of remote sensing in disease ecology, based on our quantitative review: (i) mapping host abundance and diversity, (ii) understanding host movement and mobility, (iii) early detection of disease, (iv) modelling pathogen environmental dynamics, and (v) forecasting future disease risk. For each application, we use a focal disease to identify a challenge in the use of remotely sensed data and propose possible solutions that would improve the use of remote sensing (tables 2 and 3).

**Table 2.** Applications of remote sensing in disease ecology.

disease ecology application	example disease	current challenge	possible solution
**mapping host distributions**	hantaviruses	**proxy variables** (e.g. vegetation indices) can have multiple biological interpretations	consider vegetation metrics with biological interpretation such as vegetation structure, when available
**understanding host movement**	avian influenza	**temporal resolution** of remotely sensed data does not match temporal scale of animal movement patterns	couple high temporal resolution (e.g. geostationary satellites) with higher spatial resolution (e.g. traditional multispectral sensors)
**early detection of disease**	pine wilt disease	**spatial and/or spectral resolution** of satellite data cannot detect disease signs in individual plants; UAV and airborne flights are relatively infrequent and/or unreplicated	use predictive models to combine UAV/airborne disease detection and satellite-sensed environmental data
**modelling pathogen environmental dynamics**	soil-transmitted helminths	**soil and water data** can be difficult to obtain remotely at fine enough resolutions	couple temporally static *in situ* datasets with dynamic satellite-derived data on soil moisture
**forecasting future risk**	malaria	studies often ignore **uncertainty** in remote sensing and in climate models	use provided uncertainty and/or quality layers; at a minimum, acknowledge data uncertainty as well as model uncertainty

**Table 3.** Available and upcoming satellite missions that provide disease ecology-relevant ecological variables. (This list focuses on variables identified in the literature review and on datasets with global or near-global extent with anticipated availability within the next 10 years. Some variables are available as derived products, while others have algorithms available for calculating ecological variables from raw data (see the ‘data and product availability’ column). Extent can vary across sources. Temporally static products (‘resolution’ column) are those with a single available product, usually derived from data collected across a range of dates. For more detail on each mission and product, including data access links and full names, see the electronic supplementary material, table S1.)

variable type, disease ecology application(s)	variable	data sources (sensor types, missions, and/or datasets)	extent	resolution	data and product availability
***vegetation properties*** — measure habitat suitability for wildlife hosts — monitor plant health and disease	vegetation aboveground biomass	synthetic aperture radar (SAR) missions (NISAR, ESA Biomass, ALOS PALSAR, Sentinel−1)	global; 2006 to present	10–30 m, 14 days	biomass must be calculated from backscatter; algorithms are available for some missions
	vegetation height; vegetation structure	LiDAR missions (GEDI)	52°N−52° S; 2019–2023, 2025 onwards	10 m, static	for 10 m canopy height models, see [29]. Algorithms and tutorials for LiDAR point cloud data are available
	plant functional type	narrow-range spectroscopy, hyperspectral sensors (SBG, CHIME)	global; approx. 2030 onwards	30 m, static	product is planned but not yet available
	photosynthetic activity	narrow-range spectroscopy (SBG, ESA FLEX, TROPOMI/Sentinel−5P)	global; 2017 to present	300 m–7 km, 27 days	algorithms are available for some missions to calculate vegetation fluorescence from band values
	land cover (vegetation classification, related habitat variables)	broad-range multispectral (VIIRS, Landsat, Sentinel−2, MODIS)	global; 1972 to present	10–500 m, annual-multiannual	multiple classifications are available from diverse sources. Custom classifications can also be created
	vegetation indices	broad-range multispectral (VIIRS, Landsat, Sentinel−2, MODIS)	global; 1972 to present	10–500 m, 5–16 days	a subset of indices are available as products for some sensors; algorithms are available for other indices
***high temporal resolution weather data*** — understand and forecast host dispersal events — model and map vector biting rates	rainfall, smoke, land surface temperature	geostationary satellites (GOES, Himawari, Meteosat)	hemispheric; 1975 to present	0.5–10 km, 10 min-1 h	products are available for some weather variables
	rainfall, snowfall, air temperature	multi-sensor reanalysis products (ERA5, MERRA−2)	global; 1950 to present	5 km, hourly	gridded products are available
	land surface temperature	geostationary-Low Earth Orbit hybrid products	global	30 m, hourly	in development; no products or algorithms available
***VHR optical imagery*** — detect plant diseases — measure habitat for wildlife hosts	panchromatic and multispectral imagery	multispectral, panchromatic VHR satellites (Pléiades, PlanetScope/SkySat, RapidEye, WorldView, Quickbird)	global; 2001 to present	30 cm-5 m, daily−5 days	imagery available at cost from commercial data providers; multispectral data can be processed using custom or existing algorithms (e.g. land cover, vegetation indices)
***soil parameters*** — model parasite environmental development and survival	soil moisture	SAR and microwave (SMAP, SMOS)	global; 2015 to present	9–35 km, 3–23 days	soil moisture products are available
		SAR missions (NISAR)	global; 2024 to present	200 m, 12 days	product is planned but not yet available
		broad-range spectroscopy (e.g. MODIS/Sentinel−3) combined with *in situ* data	N/A	1 km, daily	algorithms can combine data types to estimate soil moisture
	land surface temperature (LST)	thermal bands of multispectral sensors (VIIRS, MODIS, Landsat)	global; 1999 to present	100 m-1 km, 2–16 days	most multispectral missions provide LST products, which can be used to measure soil temperature
***surface water properties (extent, depth)*** — model and map host and vector distributions — model exposure to environmentally transmitted pathogens	surface water extent	SAR missions (SWOT, NISAR)	global; 2023 to present	100 m, 12–21 days	SWOT surface water extent product is available; NISAR product or algorithm is planned but not yet available
		broad-range spectroscopy (Landsat, Sentintel−2, MODIS)	global; 1984 to present	30 m, 5 days-monthly	products are available
	surface water depth	SWOT	78°S−78°N; 2023 to present	100 m, 21 days	calculate from water surface elevation and extent; algorithm not yet available

### (a) Mapping host abundance and diversity: hantaviruses and proxy variables

Host distributions govern the transmission of infectious pathogens by determining exposure of susceptible hosts to infectious hosts and contaminated environments. The use of remote sensing to understand host or vector distributions, abundance and/or diversity was very common in our review (approx. 50% of studies). This application is illustrated by mapping of hantavirus reservoir hosts (e.g. [30]). Hantaviruses are zoonoses that are transmitted to humans via contact with infected rodents or their excrement. Because there is no human-to-human transmission of hantaviruses, the overlap of rodent distributions with human populations is a primary driver of disease risk in humans.

**Figure 3.**
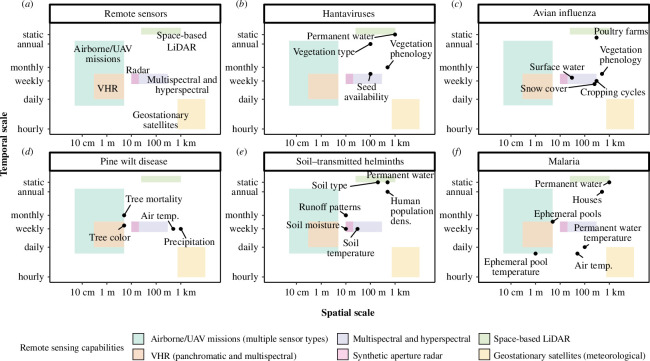
Spatio-temporal scales of key variables for infectious disease ecology. (*a*) Spatial and temporal resolutions of currently available remote sensors. Rectangles represent the range of spatial and temporal resolutions currently available from each sensor type. Resolutions can be made coarser, so rectangles represent the finest available resolutions (i.e. rectangles extend up and right). Note that different sensor types provide different variables. (*b-f*) Key variables for the study of five focal infectious diseases. Points show the ideal spatial and temporal resolution of each variable for the study of the focal disease. Points below or to the left of a coloured rectangle indicate that sufficiently fine resolutions are not yet available from a given remote sensor. For example, in panel (*e*), soil temperature at a scale relevant to the ecology of soil-transmitted helminths could not be studied using geostationary satellites alone, because spatial resolution is insufficient. Abbreviations: UAV, uncrewed aerial vehicle; DEM, digital elevation model; LiDAR, light detection and ranging; VHR, very high resolution.

Species distribution models (SDMs, also called ecological niche models or habitat suitability models) combine location data with environmental variables to estimate a host’s environmental niche and consequently its spatial distribution. In our quantitative review, use of proxy variables, most notably vegetation indices (figure 1), was common in SDMs [31,32]. Vegetation indices provide many benefits: they represent multiple ecological variables (e.g. phenology, productivity, vegetation type, land cover), are available over long time series and are commonly accepted [32]. However, this breadth trades off with precision; vegetation indices do not directly measure vegetation type or structure [33], even though this distinction can be incredibly important ecologically. For example, the enhanced vegetation index, which was the strongest predictor of hantavirus rodent reservoir abundance in Argentina, could reflect shelter, food, breeding habitat and/or protection from predators [34] (figure 3*b*). Distinguishing among these different interpretations could inform disease risk management. For instance, reducing grain waste in agricultural fields might reduce mouse abundance if mice are present owing to food availability, but not if the fields primarily provide shelter.

To advance host and vector mapping efforts, we encourage disease ecologists to consider more direct measures of habitat (table 3). For example, some land cover databases provide data on vegetation type at various spatial resolutions and levels of specificity, most commonly for forest types [35,36], which could complement or replace vegetation indices in some applications. In addition, vegetation indices can be used to distinguish between vegetation types, although these relationships are system-specific and scale-dependent and therefore require an additional validation step [37]. Other products such as canopy height models (available near-globally at 10 m resolution [29]) that measure vegetation structure could help differentiate between resource types (e.g. shelter vs forage) at relevant spatial resolutions [38]. The upcoming NASA-Indian Space Research Organisation (ISRO) Synthetic Aperture Radar (NISAR) project will quantify vegetation dynamics globally [39] and NASA’s Surface Biology and Geology (SBG) mission aims to characterize terrestrial and aquatic ecosystems, including plant functional types, using improved spectral and thermal measurements [40].

### (b) Understanding host movement and mobility: avian influenza viruses and temporal resolution

Host mobility contributes to the dispersal of pathogens across space. Because wildlife movements respond to environmental conditions, integrating remote sensing with data on animal movement can help understand drivers of pathogen dispersal. This application is illustrated by the ecology of avian influenza viruses (AIVs); migratory waterfowl are natural hosts of AIVs and their migration patterns partially determine AIV transmission [41,42]. Understanding environmental drivers of local and migratory movements at multiple spatial and temporal scales could help model and forecast host movements, animal contact patterns and AIV dispersal.

Modelling animal movement requires using environmental data at a temporal resolution that matches that of host mobility and landscape dynamics. For many species, including waterfowl, seasonal migration is driven by seasonal changes in temperature, vegetation and snow cover [43,44]. Commonly used remotely sensed vegetation and temperature products (e.g. MODIS-derived vegetation indices and land surface temperature) are available as 8- or 16 day composites, which would be suitable for measuring broad patterns in waterfowl migrations, which last 40–80 days [45]. However, in our quantitative review, other important variables such as surface water extent were often measured with land cover layers, which are updated annually or multi-annually (but see [46,47]). Furthermore, local movements of waterbirds are associated with changes in weather, forage availability and disturbances (e.g. fires, floods) that occur at hourly to daily time scales [48,49] and might not be captured by multi-day composite images (figure 3*c*).

Data at ecologically relevant high temporal resolutions can be derived from a combination of methods and sources. Geostationary (GEO) satellites provide data on land temperature, rainfall rates, smoke detection and snow cover at sub-hourly resolution (although at coarse 2 km spatial resolutions, [50]), often with fewer issues related to cloud cover compared to low Earth orbit (LEO) satellites. In the future, GEO data could be assimilated with LEO data, which are at finer spatial but coarser temporal resolutions (table 3, figure 3*a*). Synthetic aperture radar (SAR) data from current and future satellite missions such as Sentinel-1 and NISAR can also detect surface water, without interference from clouds or smoke [39] (table 3). Reanalysis products (e.g. European ReAnalysis (ERA) [51]), which combine models with observations from multiple sources, can also provide improved temporal resolution, albeit with more latency between data collection and product availability. Finally, airborne and UAV missions can monitor weather in real time, although these require advanced planning and significant resources [52].

Technological advances in animal telemetry parallel those in remote sensing, providing an emerging opportunity to understand movement-disease relationships. The most advanced modern GPS telemetry can provide data on individual animals’ locations at sub-hourly intervals with errors of less than 10 m [53,54]. Matching telemetry data with remote sensing data will therefore push the bounds of the spatio-temporal resolutions of remote sensing data [55]. Integrating these data types could facilitate management actions at fine spatial and temporal scales, such as farm-level alerts to house free-range poultry indoors in response to predicted waterfowl movements.

### (c) Early detection of pathogens and disease to facilitate management: pine wilt disease, spatial and spectral resolution

Collecting data on infections usually requires compiling case reports or performing biological sampling, but when disease causes visible clinical signs, remote sensing can be used to define a disease response (e.g. infection prevalence), in contrast to its other uses as a correlate of disease occurrence (e.g. temperature). In our quantitative review, remote sensing was used to detect plant diseases, most commonly pine wilt disease and sudden oak death. Pine wilt disease, which causes rapid wilting and mortality in some species of pine trees, is caused by a nematode parasite and spread by beetle vectors. Removal of dead and dying trees is the primary management technique for this disease, making early detection important for preventing its spread [56].

Detecting pine wilt disease requires identifying changes in colour and needle density, ideally at the level of an individual tree. Although tree diseases and pests have long been studied using remotely sensed data from Landsat (spatial resolution 30 m) and aerial imagery [57–59], high spatial resolutions (e.g. <1 m; [56]) are better suited to the spatial scale of the disease processes (figure 3*d*). Accordingly, most disease detection studies in our review relied on high-resolution airborne and UAV data sources rather than on satellites. Although it is possible to detect disease with relatively few bands [56], high spectral resolutions facilitate early detection, since changes in needle colour as the disease progresses can be subtle [60,61]. For example, PlanetScope imagery (Planet Labs), which offers multispectral daily global coverage at less than 3 m resolution, has been used to map tree mortality and to attribute mortality causes [62,63]. However, most satellite-borne sensors with very high spatial resolutions (VHR; <5 m) offer fewer spectral bands (figure 3*a*, table 3); insufficient spatial and/or spectral resolutions limit the ability to detect bark beetle infestations from remote sensing [64]. Most VHR satellites are operated by private companies with a significant acquisition cost; low- or no-cost access to such imagery would facilitate targeted studies and disease detection.

Future airborne or satellite missions that include more spectra at high spatial and temporal resolutions could help identify wilt patterns. When coupled with auxiliary environmental data on precipitation, temperature and fire, these high-resolution data could distinguish disease from other causes of mortality. In addition, because the vital rates of the beetle and nematode that cause pine wilt disease depend on ambient temperature and precipitation [65,66], models that link disease occurrence to landscape and weather data at coarser resolutions could be used to target future surveys to locales at highest risk of disease invasion [67]. Combining currently available satellite data with UAV or airborne images could also produce high-resolution time-series of disease symptoms, and SAR data could help identify symptoms that occur lower in the canopy [59]. In the more distant future, automated data collection from VHR satellites could be combined with predictive models to produce automated early detection systems for this disease, similar to established systems for *Vibrio* exposure risk in humans [10].

### (d) Modelling pathogen environmental dynamics: soil-transmitted helminths and soil properties

Pathogens with an environmental life stage depend on suitable environmental conditions for survival and/or development. Soil-transmitted helminths, many of which cause NTDs, are difficult to manage because they infect multiple hosts and persist for long periods in soil or water [68]. Although pathogen dynamics cannot be measured remotely, remotely sensed data on environmental conditions can be used to parameterize models of pathogen development based on laboratory or field measurements.

Modelling helminth development in soil requires measuring environmental conditions including soil type, soil temperature and soil moisture (figure 3*e*). Many studies in our review used datasets such as SoilGrids [69], which provides soil physical and chemical properties (e.g. pH, clay content) based on a model parameterized with *in situ* soil profile observations and environmental covariates (some remotely sensed [70]). These datasets are ideal for variables that change little over time, but not for dynamic variables like soil moisture [71], which limits researchers’ abilities to model helminth development dynamics at biologically relevant time scales.

A combination of precipitation, relative humidity and land surface temperature can serve as proxies for soil moisture (e.g. [72]), and newer satellite-based microwave sensors provide both surface and subsurface moisture data, albeit at coarse spatial resolutions (e.g. SMAP; table 3). Future remote sensing products that use higher-resolution SAR sensors could be beneficial for modelling helminths and other soil-living parasites; for example, NISAR will provide 6–12 days soil moisture data at a 200 m scale [73]. Algorithms that use data on weather and vegetation to downscale soil moisture [74] could also be beneficial for modelling helminths and other soil-living parasites, but these algorithms have not yet been applied at a global scale.

### (e) Forecasting future risk: malaria and data uncertainty

Effective public health interventions (e.g. vaccination campaigns) depend on accurate forecasts of disease risk. Malaria is a widespread mosquito-borne disease that causes hundreds of thousands of deaths annually, most notably in sub-Saharan Africa. Climate change is expected to impact malaria as shifts in temperature and precipitation induce changes in mosquito distributions, behaviour and development rates [75]. While species range shifts occur at regional to continental scales [75], risk itself is spatially constrained because mosquitoes move only short distances (hundreds of meters to a few kilometers) during their lifetimes [76] (figure 3*f*). There is also substantial uncertainty in disease risk forecasts, stemming from uncertainty in remotely sensed data on current environmental conditions, model fitting and projections of future climate patterns [77].

In our quantitative review, studies commonly reported and/or analysed model uncertainty, but few accounted for uncertainty in their underlying remotely sensed data or climate projections ([78,79] but see [80]). Many ecologists may not recognize the extent to which remote sensing data itself contains uncertainties that stem from measurement error, instrument calibration, atmospheric correction and spatial variation within a pixel [81]. Accordingly, uncertainty quantification in remotely sensed data inspires its own studies [81], but these studies require expertise in analysis of raw remote sensing data. Although many remotely sensed layers such as MODIS Land Surface Temperature provide a quality band, they do not usually directly quantify uncertainty. Because of the local nature of malaria risk, especially in urban areas [76], many studies in our review used very high-resolution (<2 m) satellite imagery to identify mosquito breeding habitats (e.g. [82]). By contrast, climate models and remotely sensed land surface temperature data are much coarser (500 m to 11 km resolution), making it difficult to project the fine-scale processes that govern mosquito breeding habitat availability. While statistical downscaling can significantly increase the spatial resolution of global climate models and mosquito distribution forecasts [75], process-based dynamical models might be a more effective avenue for regional and local forecasts of transmission risk (e.g. [83]), albeit at greater computational costs.

Uncertainty compounds as multiple data sources are combined, making it particularly important to consider uncertainty propagation across data sources as well as in model estimation. Some statistical methods are better suited than others for incorporating uncertainty; for example, state-space models can partition uncertainty between observation error and model specification and could be useful in disease forecasting because they are well suited to spatio-temporally structured data [84]. Similarly, stochastic dynamical models can simultaneously simulate variation owing to random variation and parameter uncertainty [83]. By contrast, it can be difficult to quantify uncertainty when using some common SDM methods, such as random forests. We therefore suggest that ecologists consider methods that quantify the impacts of uncertainty in underlying remotely sensed data on their results and, importantly, that they report these impacts. For example, maps could present estimates of uncertainty (e.g. confidence intervals [80]) across a range of conditions or scenarios.

## 4. Conclusions

Remote measurements of environmental variables play an important role in disease ecology by enabling mapping, forecasting and inference about drivers of infectious disease outbreaks in diverse host-pathogen systems. Many remotely sensed data products are readily available online, often at no cost. The increasing prevalence of online resources, including server-based interfaces that eliminate the need to download large datasets for analysis [85], especially when combined with training programmes (e.g. NASA ARSET [86]), are contributing to expanded use of remotely sensed data across ecological fields [85]. This review highlights that remote sensing contributes significantly to disease ecology research; continuing to increase access to remote sensing can contribute to global environmental and public health by empowering local scientists, practitioners and decision-makers to research and manage local systems [6,87].

Ongoing advances in remote sensing technology, including improvements in spatio-temporal and spectral resolutions, products measuring new variables, and reduced latency in product availability, will provide new opportunities for ecologists to gain more precise and timely understandings of natural systems. However, many ecologists lack the computational resources, technical expertise and time to process large satellite-derived datasets to create custom products [88]; translating these technological advancements into scientific ones thus requires communication and collaboration between remote sensing scientists and disease ecologists to identify needs and opportunities [89]. Such collaborations would be enabled by venues and funding opportunities that encourage participation by researchers from both groups, similar to the Group on Earth Observations Biodiversity Observation Network (GEO BON), U.S. Geological Survey’s National Land Imaging Program (USGS NLI), and ESA-Future Earth Joint Program [90–92].

For many ecological applications, data already exist to fill research gaps—instead, the need is for awareness, time and resources. For example, our quantitative review identified relatively few studies of non-zoonotic wildlife diseases, for which existing disease sampling (e.g. from disease surveillance of harvested or at-risk species) could be combined with remotely sensed environmental data. Although it is tempting to seek remotely sensed data at ever-finer spatial and temporal resolutions, these characteristics quickly drive up hardware, downlink, and processing costs, and might not always be necessary for study goals; for example, if disease data is identified only at the town or locality level, or if intervention occurs at a regional scale, remote sensing data with moderate spatial resolutions might be suitable.

In an age of rapid disease emergence [1–3], it is ever more urgent to study the ecology of infectious diseases in humans, plants and wildlife. The COVID-19 pandemic highlighted the use of remote sensing for studying both the causes and consequences of outbreaks by providing standardized, accessible environmental data at regional to global extents [93]. Alongside environmental data, it is also imperative that researchers and practitioners consider the social and behavioural determinants of disease in both human and non-human systems [94,95] and integrate remote sensing with other modern (bio)technologies [8]. Remote sensing is a powerful and increasingly important tool in the disease ecologist’s toolbox, especially when used thoughtfully and with the input of disciplinary experts.

## Data Availability

Data from the quantitative review are available from Zenodo [14]. Supplementary material is available online [96].
